# HIV serostatus disclosure is not associated with safer sexual behavior among HIV-positive men who have sex with men (MSM) and their partners at risk for infection in Bangkok, Thailand

**DOI:** 10.1186/1742-6405-9-38

**Published:** 2012-12-23

**Authors:** Nneka Edwards-Jackson, Nittaya Phanuphak, Hong Van Tieu, Nitiya Chomchey, Nipat Teeratakulpisarn, Wassana Sathienthammawit, Charnwit Pakam, Nutthasun Pharachetsakul, Magdalena E Sobieszczyk, Praphan Phanuphak, Jintanat Ananworanich

**Affiliations:** 1 College of Physicians & Surgeons, Columbia University, New York, USA; 2 SEARCH, 104 Rajdumri Road, Pathumwan, Bangkok, 10330, Thailand; 3 The Thai Red Cross AIDS Research Center, 104 Rajdumri Road, Pathumwan, Bangkok, 10330, Thailand; 4 Laboratory of Infectious Disease Prevention, Lindsley F. Kimball Research Institute, New York Blood Center, New York, USA; 5 Faculty of Medicine, Chulalongkorn University, Bangkok, Thailand

**Keywords:** HIV-positive, Serostatus disclosure, Men who have sex with men, Thailand

## Abstract

**Background:**

The relationship between HIV serostatus disclosure and sexual risk behavior is inconsistent across studies. As men who have sex with men (MSM) are emerging as the key affected population in Bangkok, Thailand with reported HIV prevalence of 30%, we assessed whether HIV disclosure is associated with protected sex in this population.

**Methods:**

A risk behavior questionnaire was administered using Audio Computer-Assisted Self-Interviewing (ACASI) to determine whether HIV serostatus disclosure was associated with protected sex in 200 HIV-positive MSM in Bangkok. HIV serostatus disclosure to the most recent sexual partner prior to or at the time of the sexual encounter was assessed. Protected sex was defined as insertive or receptive anal intercourse with a condom at the most recent sexual encounter.

**Results:**

The mean age was 30.2 years, CD4 was 353 cells/mm^3^, and one-third was on antiretroviral therapy. At the most recent sexual encounter, HIV serostatus disclosure rate was low (26%); 60.5% of subjects had not discussed their serostatus at all, while 5.5% had not revealed their true serostatus. Seventeen percent reported unprotected anal intercourse and about half had sex with their primary partners. The serostatus of the most recent sexual partner was HIV-positive in 19.2%, HIV-negative in 26.4%, and unknown in 54.4% of subjects. There was no association between disclosure and protected sex, with 41 of 48 (85.4%) disclosers and 104 of 126 (82.5%) of non-disclosers reported protected sex (p = .65). Subjects with HIV-positive partners were less likely to report protected sex overall (20 of 33, 60.6%) compared to those with HIV negative (82 of 96, 85.4%) or unknown (41 of 45, 91.1%) partners (p = .001). Age (27-32 years vs. ≤26 years, p = .008), primary partner status (p < .001), and HIV-positive serostatus of sexual partner (p < .001) were significantly associated with disclosure in the multivariate analyses.

**Conclusion:**

Rates of HIV disclosure to sexual partners by HIV-positive MSM in Bangkok are low. Despite low rates of HIV serostatus disclosure, most HIV-positive MSM reported protected sex with their partners at risk for infection. Future studies should focus on understanding barriers to disclosure and factors driving risk behavior amongst MSM in Thailand.

## Background

Men who have sex with men (MSM) have emerged as a population at high risk for HIV-1 infection in Thailand. In 2003, a community-based study among MSM in Bangkok demonstrated HIV prevalence as high as17.3% [1]. Follow-up studies showed this prevalence had increased to 28.3% in 2005 and 30.8% in 2007 [1,2]. An examination of the factors that influence the transmission of HIV in the Thai MSM community is needed to help guide prevention efforts. Disclosure of HIV serostatus to sexual partners may serve as an important prevention intervention in this population.

HIV serostatus disclosure has been well studied in Western countries. Although various factors have been shown to influence the likelihood of disclosure among HIV-positive MSM [3-5], the relationship between HIV serostatus disclosure and sexual risk behavior, has been inconsistent across studies. Some studies have revealed that disclosers are more likely to practice safer sexual behaviors [5,6] while others have shown no association [3,7].

Data are lacking on HIV serostatus disclosure among HIV-positive MSM in Thailand. We therefore aimed to determine whether HIV disclosure was associated with protected sex, and to describe the factors associated with disclosure.

## Results

Table 1 outlines the demographics and behavioral characteristics of the study population. Mean age was 30.2 years. Mean duration of known HIV infection was 3 years with a mean CD4 T cell count of 353 cells/mm^3^. About one-third of men was on antiretroviral therapy (ART). Subjects reported a mean of 3.3 male sexual partners in the past 3 months. At their most recent sexual encounter, 17.2% and 70.9% of men reported engaging in unprotected anal and oral intercourse, respectively; 7.0% and 9.0% either did not respond or denied that sexual route at their last sexual encounter, respectively. Only 26.0% of participants had disclosed their HIV-positive serostatus to their partner prior to that encounter; 60.5% of subjects had not discussed their serostatus at all, while 5.5% had not revealed their true serostatus. About half (51.1%) of the most recent sexual partners were primary partners. The serostatus of the most recent sexual partner was HIV-positive in 19.2%, HIV-negative in 26.4%, and unknown in 54.4% of subjects.

**Table 1:** Demographic and sexual behavior data (N = 200)^a^

**Demographics**	
Age, years, n (%)	
≤25	57 (28.5)
26 – 30	64 (32.0)
31 – 35	37 (18.5)
>35	42 (21.0)
Education level, n (%)	
High school and below	37 (18.5)
Vocational/technical training	25 (12.5)
College or above	134 (67.0)
Monthly income, n (%)^b^	
<10,000 Thai baht	61 (30.5)
10,000-20,000 Thai baht	81 (40.5)
>20,000 Thai baht	48 (24.0)
Occupation, n (%)	
Student	29 (14.8)
Employed	142 (72.4)
Unemployed	23 (11.7)
Retired	2 (1.0)
Current household, n (%)	
Living with spouse or primary partner	30 (15.0)
Living with roommate(s)	35 (17.5)
Living with relatives	68 (34.0)
Years since HIV diagnosis, n (%)	
0 – 1 year	93 (50.5)
2 – 4 years	52 (26.0)
5 or more years	39 (19.5)
Current CD4 count, cells/mm^3^, n (%)	
≤ 200	28 (14.0)
201 – 350	62 (31.0)
351 – 500	55 (27.5)
> 500	30 (15.0)
Current anti-retroviral use, n (%)	66 (33.0)
History of AIDS-defining illness, n (%)^c^	32 (16.0)
**Sexual identity and behavior**	
Sexual orientation, n (%)	
Homosexual	171 (85.5)
Bisexual	16 (8.0)
Uncertain of sexuality	4 (3.0)
Number of male sexual partners in the past 3 months, n (%)^d^	
1	103 (51.5)
2-5	73 (36.5)
6-10	10 (5.0)
>10	11 (5.5)
Type of most recent sexual partner^e^	
Primary or main partner	93 (51.1)
Secondary partner	89 (48.9)
HIV serostatus of most recent sexual partner	
Known HIV-positive	35 (19.2)
Known HIV-negative	48 (26.4)
Serostatus-unknown	99 (54.4)
Disclosure of HIV status to most recent sexual partner, n (%)^e^	
Disclosure of HIV-positive status	52 (26.0)
No disclosure	121 (60.5)
Disclosure of HIV-negative or unknown status	11 (5.5)
Disclosure by someone else	1 (0.5)
Partner awareness of subject’s HIV-positive status^e^	
Partner aware of HIV-positive status	56 (28.0)
Partner unaware	125 (62.5)
Uncertain of partner’s awareness	9 (4.5)
Unprotected intercourse, n (%)g	
Unprotected anal intercourse	32 (17.2)
Unprotected oral intercourse	129 (70.9)

^a^All percentages presented were calculated using N = 200 to include non-responders.

^b^Average monthly *household* income in Thailand in 2007 was 18,660 baht, or approximately 570 USD (National Statistical Office, 2008).

^c^Centers for Disease Control and Prevention Classification.

^d^No female sexual partners were reported.

^e^Primary or main partners were partners whom one lives with or spends a lot of time with, and to whom one feels an emotional commitment. Secondary partners were all non-primary sexual partners.

^f^Partner awareness was assessed by the question, “Did your *most recent sexual partner* know you are infected with HIV *before you had sex*?”. Disclosure was assessed with the follow-up question, “Did you discuss your HIV status with this person *before you had sex*?”.

Reported rates of protected anal intercourse were relatively high, but there was no association between disclosure and protected sex; 41 of 48 (85.4%) disclosers and 104 of 126 (82.5%) of non-disclosers reported protected sex (p = 0.65; OR 1.23, 95% CI [0.49, 3.12]; adjusted OR 1.58, 95% CI [0.47, 5.35]) (Table 2). The most common behavioral pattern of disclosure and sexual behavior was non-disclosure/protected sex, which was reported by 60.0% of subjects. The proportions of subjects who reported disclosure/protected sex, disclosure/unprotected sex, and non-disclosure/unprotected sex were 23.6%, 4.0%, and 12.6%, respectively.

**Table 2:** Relationship between HIV serostatus disclosure and protected sex^a^

	**Disclosers N (%)**	**Non-disclosers N (%)**	**p**	**OR (95% CI)**	**Adjusted OR (95% CI)**
Overall (N = 174)	41 (85.4)	104 (82.5)	0.65	1.23 (0.49, 3.12)	1.58 (0.47, 5.35)^b^
Subjects with HIV-positive partners (N = 32)	22 (77.3)	2 (20.0)	0.005	13.6 (2.15, 85.9)	13.0 (1.28, 125)^c^
Subjects with HIV-negative partners (N = 41)	10 (100)	30 (96.8)	0.76	__ ^d^	__^d^
Subjects with HIV-unknown partners (N = 91)	10 (90.0)	67 (83.8)	0.54	1.94 (0.23, 16.5)	1.74 (0.20, 18.2)^e^

^a^Includes insertive or receptive anal intercourse at the most recent sexual encounter.

^b^Adjusted for age, alcohol and drug use at last sexual encounter, and years since HIV diagnosis.

^c^Adjusted for partner type and alcohol and drug use at last sexual encounter.

^d^Not calculated due to small number of subjects engaging in unprotected sex.

^e^Adjusted for age and alcohol and drug use at last sexual encounter.

Among subjects with HIV-positive partners (n = 32), disclosers were more likely to report protected sex than non-disclosers; 17 of 22 (77.3%) disclosers versus 2 out of 10 (20.0%) non-disclosers reported protected sex (p = 0.005; OR 13.6, 95% CI [2.15, 85.9]; adjusted OR 13.0, 95% CI [1.28, 125]) (Table 2). There was no association between disclosure and protected sex among subjects with serodiscordant and serostatus-unknown partners as most of these men reported protected sex with partners regardless of whether or not they disclosed. Among subjects with HIV-negative partners, 10 of 10 (100%) disclosers reported protected sex versus 30 of 31 (96.8%) of non-disclosers (p = 0.76, odds ratios not calculated due to small number of subjects engaging in unprotected sex). Among subjects whose partner’s HIV serostatus was unknown, 10 of 11 (90.0%) disclosers reported protected sex versus 67 of 80 (83.8%) of non-disclosers (p = 0.54; OR 1.94, 95% CI [0.23, 16.5]; adjusted OR 1.74, 95% CI [0.20, 18.2]) (Table 2). Of note, subjects with HIV-positive partners were less likely to report protected sex overall (20 of 33, 60.6%) compared to those with HIV negative (82 of 96, 85.4%) or unknown (41 of 45, 91.1%) partners (p = .001). There was no association between disclosure and protected sex among other subgroups of age or partner type.

Of the factors examined (age, partner type and HIV serostatus, number of sexual partners in the past 3 months, sexual orientation and level of outness [8], alcohol and drug use at last sexual encounter, years since HIV diagnosis, CD4 count, current antiretroviral use, history of AIDS diagnoses, and level of HIV knowledge [9]), only age (27-32 years vs. ≤26 years, p = 0.008, adjusted OR 6.21 95% CI [2.0, 19.6]), primary partner status (p < 0.001, adjusted OR 16.6 95% CI [5.0, 55.5]), and HIV-positive serostatus of sexual partner (p < 0.001, adjusted OR 13.6 95% CI [4.3, 42.4]) were significantly associated with disclosure in the multivariate analyses.

## Discussion

In this study, we demonstrated low rates of HIV serostatus disclosure to sexual partners by HIV-positive MSM in Bangkok. Factors associated with HIV serostatus disclosure included older age, primary partner status, and HIV-positive serostatus of sexual partner. There was no significant association between disclosure of HIV serostatus and self-reported protected sex except in men who have HIV positive partners. Furthermore, despite a lack of disclosure, most HIV-positive men reported protected sex with serodiscordant and serostatus-unknown partners.

The rate of HIV disclosure in our cohort was low (26%). Similar to studies in other countries [4], men whose most recent sexual partner was either a non-primary or serodiscordant partner had the lowest rates of disclosure. We also found that younger men were less likely to disclose. Previous studies have found positive associations between HIV serostatus disclosure with other factors such as sexual orientation and HIV disease stage [4,5]; we did not find such associations in this study.

Since we did not explore the reasons for non-disclosure, it remains unclear why so few men in our study population chose to disclose. There could be multiple barriers to disclosing one’s HIV serostatus to sexual partners, such as fear of rejection, lack of trust or intimacy with sexual partner, fear of stigmatization or discrimination, lack of self-efficacy for disclosure, or lack of feelings of personal responsibility to disclose, especially if protected sex is planned.

A major finding of this study was the general lack of an association between HIV serostatus disclosure and protected sex. The most commonly reported behavioral pattern in our population was to withhold disclosure while practicing protected sex. This “uniformed protection” may represent a compromise between a fear of negative consequences of disclosure (e.g., refusal to have sex, stigmatization, discrimination, loss of privacy) and a desire to not put one’s sexual partner at risk [7], thereby highlighting the challenges associated with serostatus disclosure.

There were two interesting findings among men in seroconcordant relationships. As a group, men with a seroconcordant partner were less likely to have protected sex than men with a serodiscordant or serostatus-unknown partner. But when they did report protected sex, it was associated with disclosure. This suggests that men in this group were less aware of or less concerned about risks such as HIV superinfection and transmission of drug resistant virus and thereby did not prioritize using condoms or mutually disclosing their HIV-positive serostatus.

This study has several limitations. First, the study population of clients who sought HIV testing at our center may not be representative of the general MSM population in Bangkok. Second, we only analyzed sexual behavior and disclosure practices with the most recent sexual partner, instead of examining these practices across multiple partners and sexual encounters. However, our strategy may have increased the reliability of subject responses and decreased recall bias [7]. Finally, we were limited by missing data, which accounted for a percentage of non-responders for most variables; however, the response rate to all of these variables was 88% or greater.

## Conclusions

In conclusion, rates of HIV disclosure to sexual partners by HIV-positive MSM in Bangkok are low. Despite low rates of HIV serostatus disclosure, most HIV-positive MSM reported protected sex with their partners at risk for infection. Future studies should focus on understanding the barriers to discussion of serostatus amongst MSM and the factors that continue to drive unprotected sex in this population.

## Methods

This was a cross-sectional questionnaire survey among 200 HIV-positive MSM receiving services from the Thai Red Cross Anonymous Clinic, an HIV voluntary counseling and testing center in Bangkok, Thailand. Inclusion criteria included male gender at birth, ≥ 18 years of age, HIV-positive serostatus confirmed on prior testing, reporting either insertive and/or receptive anal sex with a male in the past 3 months, able to speak and read Thai, and having basic computer skills. The study protocol was approved by the Chulalongkorn University and Columbia University Institutional Review Boards. Eligible men were identified and recruited by the study nurse during their follow-up clinic appointments or initial walk-in visits at the Thai Red Cross Anonymous Clinic. Men were asked to complete a 20-minute behavioral questionnaire in Thai using Audio Computer-Assisted Self-Interviewing. Most recent CD4 T cell count (drawn at that visit or at the most recent visit prior to enrollment) was extracted from the clinic’s laboratory database. Data collection occurred between March and June of 2010.

HIV serostatus disclosure to the most recent sexual partner prior to or at the time of sexual encounter was assessed by asking the question, “Did your *most recent sexual partner* know you are infected with HIV *before you had sex*?” (to assess partner awareness), followed by the question, “Did you discuss your HIV status with this person *before you had sex*?” An answer of “Yes, I told him/her I was HIV positive” was classified as disclosure; any other response was considered non-disclosure. Protected sex was defined as insertive or receptive anal intercourse with a condom at the most recent sexual encounter. Chi-square and Fisher exact tests were used in analyses to evaluate the association between protected sex and disclosure (with protected sex as the dependent variable) and the factors associated with disclosure (with disclosure as the dependent variable). Binary logistic regression models were developed, including covariates with p < 0.15 in the univariate analyses; adjusted odds ratios (OR) with 95% confidence intervals (CI) were calculated. Statistical analysis was conducted with SPSS Version X (SPSS Inc, Chicago, IL, USA).

## Abbreviations

ART: Antiretroviral therapy; MSM: Men who have sex with men.

## Competing interests

The authors declare that they have no competing interests.

## Authors’ contributions

NEJ, Nittaya P, HVT, MES, PP and JA designed the study. NEJ, Nittaya P and JA drafted the protocol and the manuscript. NEJ, NC, NT, WS, CP, Nutthasun P carried out the study. NEJ performed the statistical analysis. All authors read, gave input and approved the final manuscript.
